# Editorial: Gilles de la Tourette Syndrome: Cross-Cultural Perspectives With a Focus on the Asia-Pacific Region

**DOI:** 10.3389/fpsyt.2021.678089

**Published:** 2021-04-12

**Authors:** Valsamma Eapen

**Affiliations:** South Western Sydney Local Health District, Sydney, NSW, Australia

**Keywords:** Gilles de la Tourette syndrome, OCD, neurodevelomental disorders, tic discorder, Asia - Pacific

Gilles de la Tourette Syndrome (GTS) is a neurodevelopmental disorder characterized by the presence of multiple motor and one or more vocal tics of more than one-year duration (1). Most people with GTS also experience associated psychiatric co-morbidities and other challenges, leading to significant social impact and poor quality of life (2). Once thought to be rare, tic disorders are now recognized to be relatively common, with an estimated prevalence of 1% in children and adolescents in the majority of cultures of the world (1). However, GTS is often under-recognized, and a significant cause of hidden disability. Epidemiological, phenomenological, and genetic studies have demonstrated broad overlap between GTS and commonly occurring co-morbidities such as Obsessive-Compulsive Disorder (OCD) and Attention Deficit Hyperactivity Disorder (ADHD) and less commonly Autism Spectrum Disorder (3–5). Further, it has been proposed that OCD may represent an alternative phenotypic expression of the putative GTS genes with a gender-dependent difference in expression in that male family members typically present with tics while female members present more often with OCD (6). Recent research has also shown that only around one in ten patients with GTS have “pure” GTS with only motor and vocal tics, while the rest have a number of co-morbidities (7). The precise mechanisms underlying GTS are yet to be revealed, however, research suggest the involvement of a number of neurodevelopmental genes and the neurexin trans-synaptic connexus (NTSC) (8).

While GTS presents in all ethnic and cultural groups worldwide, there has been an overrepresentation of GTS literature from European and North American perspectives. However, given the heterogenous nature of the disorder, the influence of race, culture and environmental factors on symptom expression merits further exploration; the focus of this special issue. In this regard, it has been shown that, while GTS is seen less frequently in some cultures, in all cultures where it has been reported, the phenomenology is similar, highlighting the biological underpinnings of the disorder while there is also some evidence to suggest there may be some variations in the occurrence of associated behaviors and co-morbidities (9). A study of clinical patients from the UK and the United Arab Emirates for example found the rates of occurrence of OCD and ADHD to be similar in the two cohorts thereby emphasizing the biological and genetic link with these conditions but a much higher prevalence of Oppositional Defiant Disorder and Conduct Disorder in the UK sample suggesting the role of environmental and cultural differences in the occurrence of such co-morbidities (10). Further, cultural differences have been observed in the level of distress and dysfunction caused by GTS with Caucasian patients exhibiting less distress and impairment than in Latinos (11). It is therefore important to elucidate the differences in clinical presentation, impact of symptoms and the range of treatments that may be effective—some of which are more or less acceptable in different settings. Thus, cross-cultural perspectives and research have an important place in both helping clinicians work with people with GTS in specific cultural settings, but also in deepening our understanding of the disorder by observing it within different contexts as illustrated in this special issue.

Regarding phenomenology, Matsuda et al. in their study have examined whether awareness of premonitory sensation could assist in tic suppression and explored the relationship between tic-associated sensation and the potential subtypes or variants of such sensation. Further, they developed the “Rumination and Awareness Scale for tic-associated sensations” (RASTS), a new questionnaire to assess the intensity of the somatosensory hyperawareness and the patient's ability to identify signals of emerging tics. The findings indicate that aversive tic-associated somatosensory experiences do not facilitate tic suppression but the awareness of when the tic will occur prior to them experiencing the tic was correlated with the ability to suppress tics. Age-wise analysis found that the correlation between the premonitory awareness and tic suppression was significant only in children with GTS. The authors propose the possibility that premonitory awareness may gain aversive valence only in adulthood, for those where tic symptoms persist. This may have clinical implications in the behavioral management of tics.

In a 4 year follow up study of GTS, Kano et al. found that tics and associated sensory phenomena as well as global functioning had not changed much in this period but they observed significant improvement in obsessive compulsive symptoms (OCS). They also reported significant correlation between sensory phenomena and past symptoms of tics and OCS. The sample size being small, the results need replication.

Liu et al. has reported on the Expert Consensus on Diagnosis and Treatment of Tic Disorders in China, developed by the Chinese Child Neurology Society (CCNS). This provides a comprehensive account on the approach to clinical diagnosis of Tic Disorders along with therapeutic options such as educational, psychological, and pharmacological interventions, including traditional Chinese medicine and acupuncture.

Most genome-wide association studies (GWAS) have been carried out in populations of European descent leading to a disparity in our understanding of the genetics of complex traits between populations. For many conditions with complex genetic traits, gene regulation is extremely critical. While it is well-known the consistent enrichment of regulatory variants among trait-associated variants, the exact effects of these key variants across populations is unclear. In this regard, Yuan et al. has described higher variant allele frequency of CLCN2 G161S genotype in patients with tics and GTS compared to control population in Chinese Han population. However, this variant has not been reported in the currently available public databases, suggesting this may be a population specific finding.

Lou et al. functional alterations between GTS and healthy children in Chinese population using frequency-specific regional homogeneity (ReHo) and found that GTS patients showed decreased ReHo in the right operculum, increased ReHo in the left precentral gyrus and increased connectivity of the right superior frontal gyrus within the left executive control network. In addition, a significantly negative correlation was found between Yale Global Tic Severity Scale (YGTSS) vocal score and ReHo values of the right operculum in the highest frequency bands, while a significant positive correlation was found between YGTSS motor score and altered connectivity of the right superior frontal gyrus. The study also observed altered connectivity within the executive control network of GTS children alongside frequency-specific abnormal alterations of ReHo in the whole brain. Further research is indicated to examine the neural importance and clinical applications of these findings.

Zhao et al. have reported on an exploratory pilot study of Fecal microbiota transplantation and suggested that this helped shift the composition of the gut microbiota with restoration of B.coprocola which in turn correlated with tic symptom improvement. However, given the small sample size of this study, further research in this area is indicated before any conclusions can be made.

Clarke et al. in their paper using pathway analysis has implicated mitochondrial dynamics, structure and function (MDSF) in GTS which in turn has a role in neuronal circuitry development, synaptic connectivity, and neurotransmission. Given the sensitivity and responsiveness of mitochondria to environmental cues and their intimate role in neuronal development and function, they may be instrumental in mediating the nature of phenotypic presentation and the degree of phenotypic penetrance.

Thus, while genetic and biological factors are critical in conferring the risk to the development of GTS, environmental, and cultural factors may impact on how the tics and associated behaviors present, and are perceived, the rate of occurrence of associated co-morbidities, as well as the help seeking and referral options.

## Author Contributions

VE has conceptualized and wrote the manuscript.

## Conflict of Interest

The author declares that the research was conducted in the absence of any commercial or financial relationships that could be construed as a potential conflict of interest.
